# Risk of secondary immune thrombocytopenia following alemtuzumab treatment for multiple sclerosis: a systematic review and meta-analysis

**DOI:** 10.3389/fneur.2024.1375615

**Published:** 2024-04-10

**Authors:** Yuying Sun, Zhimei Liu, Jianguo Yang, Qingqing Jia, Jinglong Sun, Lei Wang, Fengjiao Liang, Shiyuan Song, Kaixi Wang, Xia Zhou

**Affiliations:** ^1^Shandong University of Traditional Chinese Medicine, Jinan, China; ^2^The Second Affiliated Hospital of Shandong University of Traditional Chinese Medicine, Jinan, China

**Keywords:** secondary immune thrombocytopenia, secondary autoimmune events, alemtuzumab, multiple sclerosis, adverse events, meta-analysis, immune thrombocytopenia

## Abstract

**Object:**

The purpose of this study was to evaluate the risk of secondary immune thrombocytopenia in multiple sclerosis patients treated with alemtuzumab through a meta-analysis.

**Methods:**

We searched databases including PubMed, Web of Science, OVID and EMBASE for studies reporting changes in platelet levels in MS patients treated with alemtuzumab from their inception until May 2023 and performed a meta-analysis. Information and data were screened and extracted by two researchers. The inclusion and exclusion criteria were established according to the PICOS principle. The obtained data were analyzed using the R software meta package and the quality assessment was conducted using Newcastle-Ottawa Scale (NOS). The causes of heterogeneity were analyzed using subgroup analysis and sensitivity analysis. Publication bias was evaluated using funnel plots and Egger test.

**Results:**

A total of 15 studies were included, encompassing 1,729 multiple sclerosis patients. Meta-analysis of overall secondary ITP in the included studies yielded a pooled rate of 0.0243. The overall incidence of secondary autoimmune events was 0.2589. In addition, subgroup analysis was applied using study regions and study types. The results showed that the incidence rate of secondary ITP in Europe was about 0.0207, while the incidence of autoimmune events (AEs) was 0.2158. The incidence rate of secondary ITP and AEs in North America was significantly higher than in Europe, being 0.0352 and 0.2622. And the analysis showed that the incidence rates of secondary ITP and AEs in prospective studies were 0.0391 and 0.1771. Retrospective studies had an incidence rate of secondary ITP at 2.16, and an incidence rate of AEs at 0.2743.

**Conclusion:**

This study found that there was a certain incidence of Immune thrombocytopenia in multiple sclerosis patients after treatment with alemtuzumab. Alemtuzumab may have some interference with platelet levels, and the mechanism may be associated with Treg cells. But due to the absence of a control group in the included literature, we cannot determine the specific impact of Alemtuzumab on platelet levels in patients with MS. Therefore, clinical physicians should perform a comprehensive assessment of the patient’s benefit-to-risk ratio before initiating alemtuzumab.

**Systematic Review Registration:**

Inplasy website, DOI number is 10.37766/inplasy2024.3.0007.

## 1 Introduction

Multiple sclerosis (MS) is a chronic demyelinating disease of the central nervous system, mediated by the immune system. It can damage the protective sheath that surrounds nerve fibers, leading to a disruption of electrical impulse conduction. By interfering with the brain and spinal cord, it affects bodily functions and causes a wide range of neurological symptoms such as dizziness, blurred vision and limb weakness. It is the leading non-traumatic cause of neurological disability among young and middle-aged adults (1, 2). According to statistics, there are approximately 2.8 million people worldwide living with multiple sclerosis, with an estimated one person being diagnosed every 5 min. Relapsing–remitting multiple sclerosis accounts for 80% of cases within the spectrum of MS (3).

In the past 20 years, the most significant advancement in the treatment of MS has been the development of Immunomodulatory Therapies (IMT). Numerous effective modifying drugs have been approved for clinical use, but they also come with serious adverse reactions. For example, beta-interferon (IFN-b), the first class of modified agents used to treat MS, has been associated with obvious adverse reactions such as flu-like symptoms, elevated liver enzymes, thyroid abnormalities, decreased white blood cell counts, or anemia (4). Natalizumab is the inaugural biologic agent endorsed by the Food and Drug Administration (FDA) for the management of MS and has demonstrated significant clinical efficacy. However, its primary safety concern is progressive multifocal leukoencephalopathy (PML), a serious and potentially fatal opportunistic brain infection caused by JC virus reactivation. This concern led to the drug’s temporary withdrawal from the market during its application process (4).

Alemtuzumab (ALZ) is also among the drugs approved by the FDA for the treatment of MS, and it has demonstrated favorable clinical efficacy. ALZ is a recombinant humanized monoclonal antibody that selectively binds to CD52, a glycosylated phosphatidylinositol-linked protein composed of 12 amino acids. CD52 is expressed on the surface of lymphocytes, monocytes, macrophages, eosinophils and NK cells. Quantitative analysis showed that memory B cells and myeloid dendritic cells (mDCs) display the highest number while natural killer (NK) cells, plasmacytoid dendritic cells (pDCs) and basophils have the lowest number of CD52 molecules per cell amongst lymphoid and myeloid cell populations, respectively (5). ALZ selectively binds to the antigens on the surface of circulating cells, inducing rapid depletion of lymphocyte T and B cells, and destroying the immune cells that could trigger excessive attack and immune response (6). After depletion, lymphocytes re-proliferate, allowing the body to rebuild immunity. Each infusion causes pan-lymphocyte depletion, and the speed and extent of lymphocyte subpopulation reconstruction can lead to changes in the lymphocyte lineage that persist for several years (7). Due to its selective action, ALZ has minor effect on lymphocytes in other organs such as the spleen, and in particular the preservation of lymphocytes in the bone marrow allows for possible immunoreconstitution in later stages (8). Based on this mechanism of action, ALZ is considered a promising option for the treatment of MS and was approved in 2014 as a first-or-second-line treatment for RRMS (9).

However, use of ALZ has been limited due to reports of serious adverse events following its approval. In 2019, the European Medicines Agency (EMA) reviewed its prescribing information and ultimately allowed its use as a second-line treatment for rapidly progressing adult patients and highly active disease (10). In 2021, Antonio Riccardo Buonomo et al. conducted a meta-analysis on the complications of infection following ALZ treatment for MS (11). The results estimated an incidence rate of 24% for infectious diseases and a disease rate of 6% for severe infections. Scappaticcio et al. (12) found that the incidence of inducible autoimmune thyroid events (ATEs) was as high as 33% in MS patients treated with ALZ. No other systematic review and meta-analysis of adverse events associated with ALZ versus MS has been reported. Blood disorders are another important adverse reaction that can occur after treatment with ALZ for MS, in addition to thyroid disease. ITP was one of the possible adverse events labeled in the early use of ALZ. Furthermore, Reda et al. (13) observed a higher incidence of ITP than anticipated in a cohort of patients with relapsed/refractory chronic lymphocytic leukemia (CLL) undergoing continuous low-dose alemtuzumab therapy. Although related drug experiments were conducted in the initial use of drugs, patients with MS treated with ALZ in clinical practice are on average older, have a longer course of the disease and a higher disability rate than patients in clinical trials (14, 15). Thus, the difference in demographic and clinical characteristics between clinical trials and real-world patients has emphasized the necessity of evaluating the outcomes of using ALZ in a broader and more heterogeneous population. So far, although some scholars have conducted systematic reviews and meta-analyses on secondary thyroid disease and infective complications, there are limited observational research data on the safety of ALZ intervention for the subsequent development of ITP in MS patients (16). Severe ITP can even lead to intracranial hemorrhage and endanger life, but relevant data analysis is lacking. Therefore, the collection, integration, and analysis of real-world data are essential for the rational use of ALZ in clinical practice.

Based on real clinical studies, this article provides an integrated analysis and systematic evaluation of the secondary ITP and changes in platelet levels in MS patients. It aims to document and aggregate a larger number of safety events to provide a reliable reference for clinical application, treatment and prevention of MS using ALZ, as well as to evaluate the use of ALZ in a scientifically-based manner.

## 2 Method

### 2.1 Date sources and search strategy

We conducted a comprehensive search of the databases PubMed, Web of Science, OVID and EMBASE up to May 2023. There were no restrictions on age, gender, or country during the search. The search strategy was developed based on the PICOS principles with search terms including Alemtuzumab, multiple sclerosis, immuno-thrombocytopenia, and other relevant keywords. Comprehensive search strategies for each database are detailed in the Supplementary materials. The results of the search were imported into EndNote X9.1 for further analysis and review.

### 2.2 Inclusion and exclusion criteria

Inclusion criteria: (1) The diagnosis of MS according to the McDonald’s criteria (17). (2) The patients with MS were treated with ALZ on a regular basis. (3) The platelet levels were monitored during the course of treatment. (4) The language of the article is English.

Exclusion criteria: Studies that conducted a re-analysis of previously published data, including meta-analyses and studies with duplicated data, Literature reviews, case reports, commentaries, letters, and meeting abstracts were excluded.

### 2.3 Data extraction

Two researchers conducted a full-text search, preliminary screening, and data extraction of literature that met the inclusion criteria. The data extraction tables included the following information: first author, year of publication, diagnostic criteria, age, sex, follow-up period, country or region, number of patients meeting the inclusion criteria, number of patients experiencing ITP events, and number of patients with autoimmune events. Any disagreements were resolved through discussion and negotiation between the authors involved in the study. If the two researchers could not reach a consensus, a third researcher was consulted. If necessary, we attempted to contact the authors for additional information.

### 2.4 Quality assessment

The Newcastle-Ottawa Scale (NOS) was used to assess the quality and the bias of the included studies.^1^ Studies with a NOS scores greater than 5 were included in the meta-analysis. The NOS scale was used to evaluate publication bias in the domains of selectivity, comparability, and outcome. Each study was independently assessed for bias by at least two reviewers, with a third reviewer involved in rare cases to resolve potential discrepancies. The use of this double-blind evaluation method helps to maintain objectivity and reduce the impact of individual preferences.

### 2.5 Data analysis

The meta-analysis was conducted using the meta package in R 4.2.2 software. Initially, the data was transformed using logarithmic transformation, logit transformation, arcsine transformation, and Freeman-Tukey double arcsine transformation based on the original rate. The Shapiro–Wilk normality test was performed to assess the normality of each data set. The selection of the appropriate data transformation method was based on the normal distribution demonstrated by the datasets. Subsequently, the variance estimation and calculation of confidence interval for secondary ITP and AEs in MS patients treated with ALZ were both conducted using the inverse variance method.

Heterogeneity among the included studies was evaluated using the Cochrane Q test and the I^2^ statistic. A *p*-value ≤0.05 or *I*^2^ ≥ 50% from the Cochrane Q test indicated significant variability among the studies. In the presence of significant heterogeneity, a sequential omission analysis was conducted for individual studies to assess the stability of the pooled results and to examine whether the observed heterogeneity could be eliminated. Alternatively, subgroup analysis was considered to investigate whether heterogeneity decreases among different subgroups, and the Q-Profile method was utilized to assessing interactions among subgroups. If heterogeneity cannot be eliminated, the pooled rates and their associated 95% CIs are calculated using a random-effects model. The sources of heterogeneity are carefully analyzed. The Publication bias of the included studies was evaluated using a funnel plot and Egger’s test. According to the results of the sequential omission analysis, studies with significant heterogeneity will be excluded.

## 3 Results

### 3.1 Characteristics of the studies included in the meta-analysis

**Figure 1:**
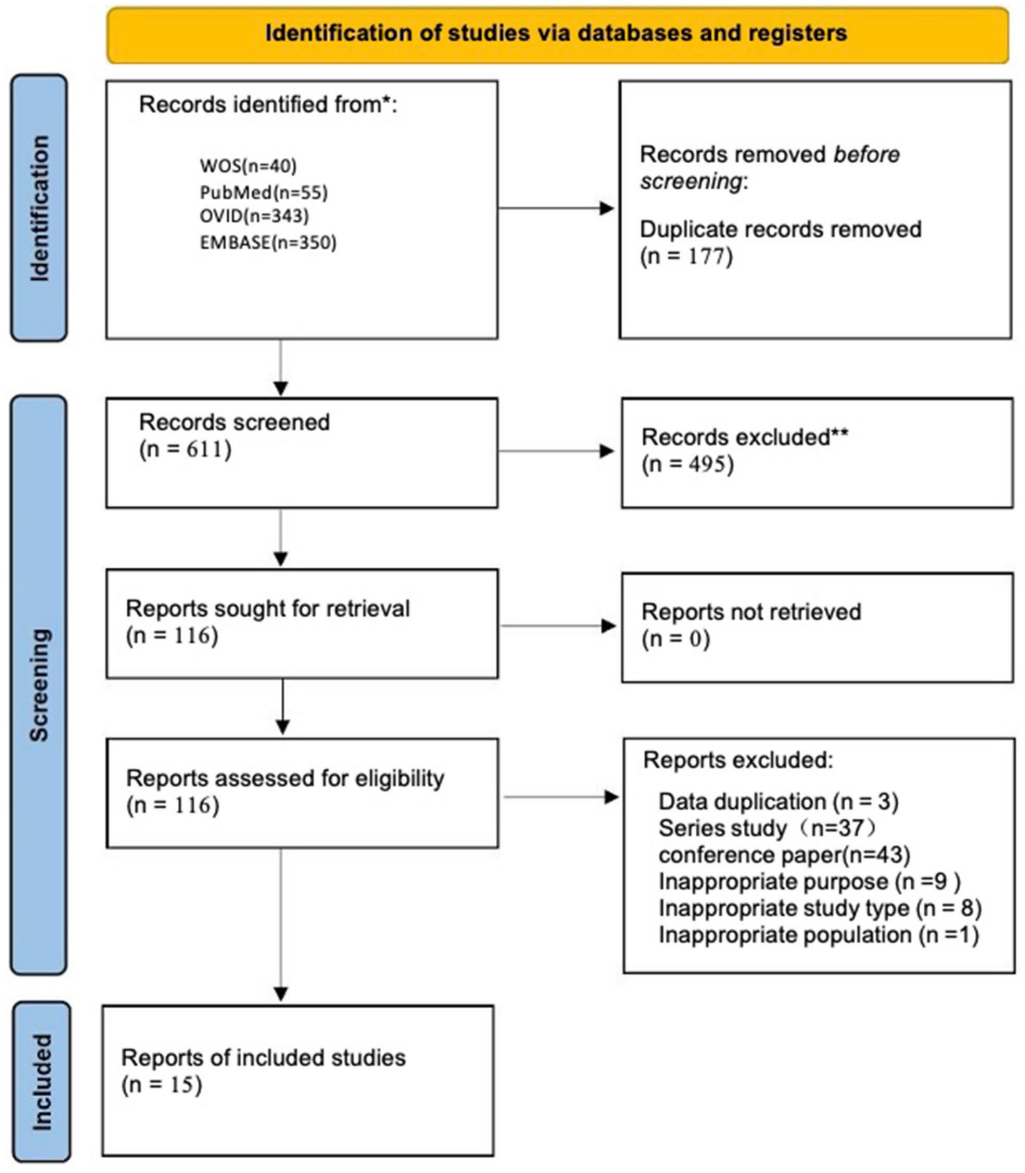
Flow chart of study selection process.

**Figure 2:**
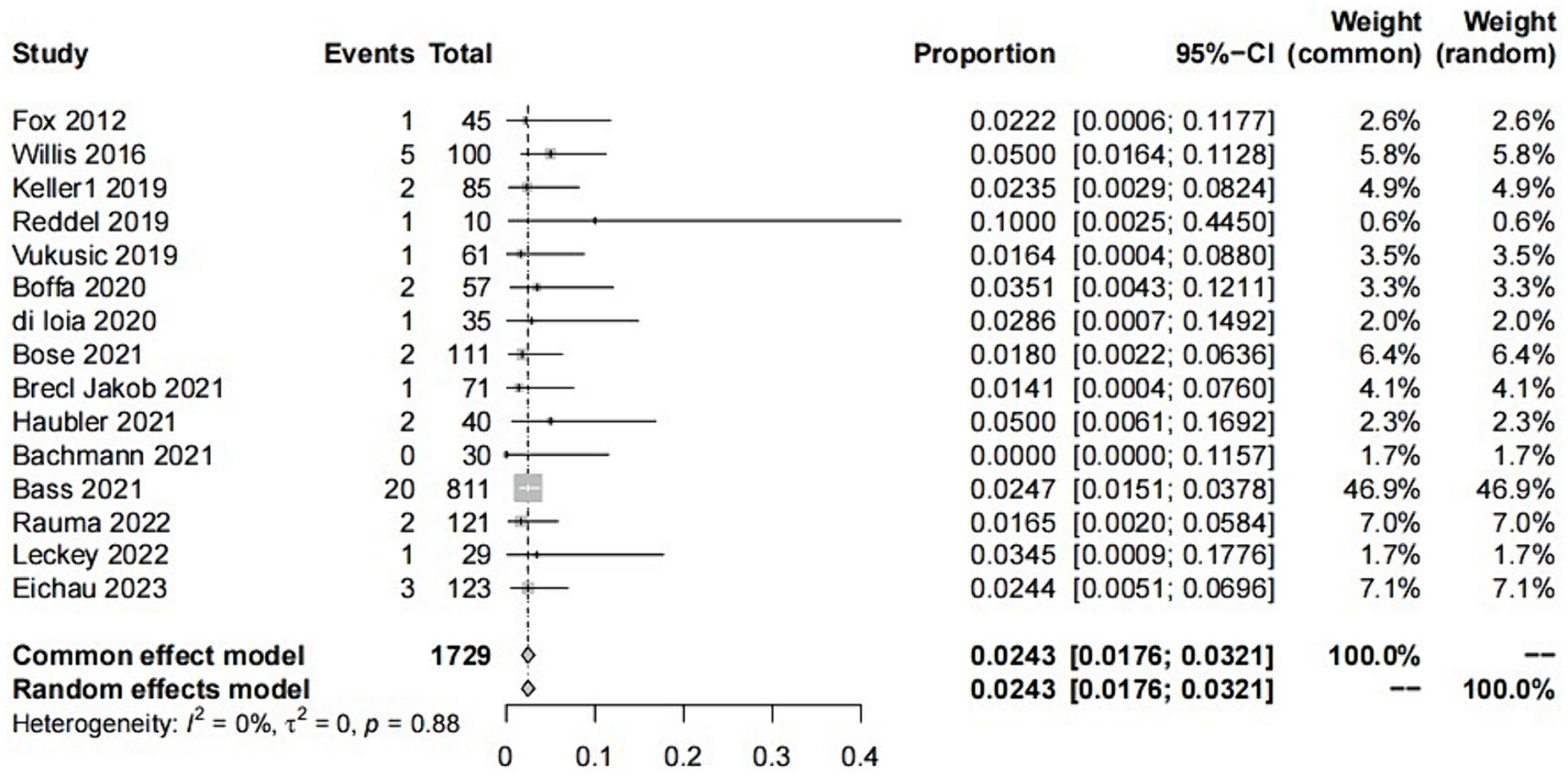
Forest of plot ITP events.

After retrieving a total of 788 articles from the database, duplicate literature found through searches in different databases and ineligible articles were removed using both software and manual methods. As a result, 116 articles written in English were retained. Of the 116 articles, 43 were conference studies, 37 were series studies, 9 did not meet the study objectives, 8 were not in the appropriate study type, 1 did not meet the subject criteria, and 3 had duplicated data, all of which were excluded (Figures 1, 2).

Finally, after analysis, 15 studies remained that met the inclusion criteria (6, 16, 18–30).

Table 1 shows that the 15 included articles were mainly published between 2019 and 2023, with two exceptions published in 2012 and 2016, respectively. The publication regions covered Europe, North America, and Oceania, with one study encompassing a nationwide scope. The majority of patients included in the studies were diagnosed with RRMS, with one study including a small proportion of SPMS patients. The diagnostic criteria are the internationally standardized Macdonald criteria. Among the 15 studies, 6 were retrospective analyses and 9 were prospective studies, with follow-up of more than 1 year in all studies (Table 2). The quality assessment using the NOS indicated scores of ≥5 for all studies, further details of which are presented in the Supplementary materials (Supplementary Table 1).

**Table 1:** Summary of main characteristics of the 15 articles included in the meta-analysis.

First author (year)	Study	Country	Cohort(n)	Cases(ITP, n)	Cases (Thro, n)	Cases (AEs, n)	Quality score
Fox et al. (30)	Prospective	USA	45	0	1	5	7
Willis et al. (6)	Prospective	USA	100	3	2	47	7
Keller et al. (29)	Prospective	Germany	85	2	0	8	7
Reddel et al. (28)	Prospective	Astralia	10	1	0	3	7
Vukusic et al. (27)	Prospective	Europe	61	1	0	4	6
Boffa et al. (26)	Prospective	Italy	57	2	0	14	8
di Ioia et al. (25)	Retrospective	Italy	35	1	0	14	6
Bose et al. (23)	Retrospective	Canada	111	2	0	28	8
Brecl Jakob et al. (22)	Retrospective	Slovenia; Croatia	71	1	0	23	7
Häußler et al. (21)	Prospective	France	40	2	0	6	8
Bachmann et al. (24)	Retrospective	Belgium	30	0	20	–	7
Bass et al. (20)	Prospective	Multicenter	811	20	0	386	7
Rauma et al. (19)	Retrospective	Finland	121	2	0	37	7
Leckey et al. (18)	Prospective	Canada	29	1	0	–	6
Eichau et al. (16)	Retrospective	Spain	123	3	0	27	7

Cohort, number of patients treated with ALZ; ALZ, alemtuzumab; ITP, immune thrombocytopenia; Thro, Thrombocytopenia; AEs, Autoimmune events.

**Table 2:** Subgroup analysis.

	Rate (ITP)	*I*^2^ (ITP)	P (ITP)	Rate (AEs)	*I*^2^ (AEs)	P (AEs)
Area						
Europe	0.0207	0%	0.80	0.2158	85%	<0.01
NA	0.0352	0%	0.62	0.2622	90%	<0.01
Study type						
Pre	0.0391	0%	0.88	0.1771	88%	<0.01
Retro	0.0216	0%	0.74	0.2743	35%	0.19

In summary, the articles are primarily published in authoritative journals and mostly belong to recent years, providing an excellent representation of the current progress and developments in the field of interest.

**Figure 3:**
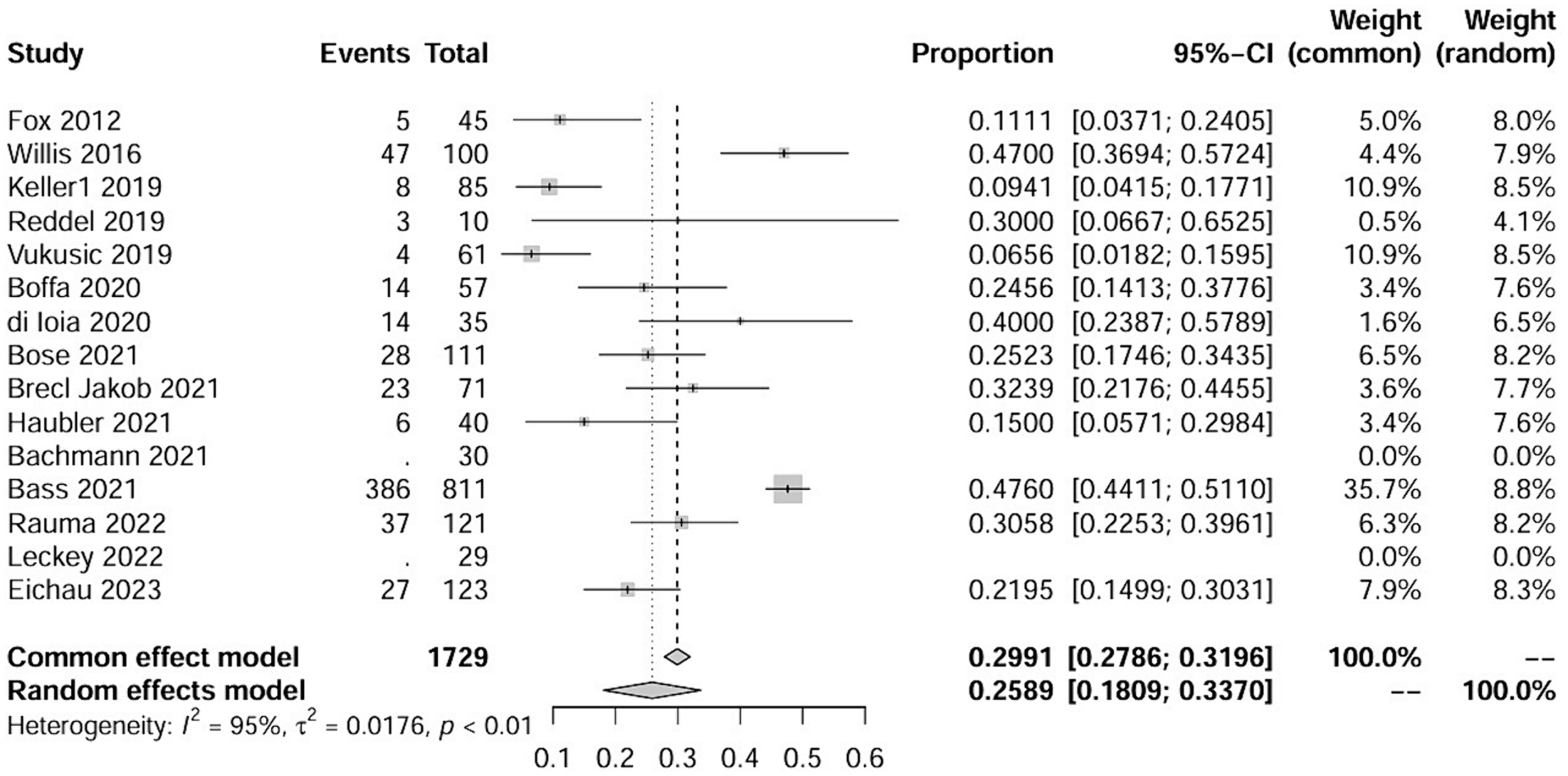
Forest plot of AEs events.

In addition, our study involved some global multicenter cohort studies: such as CARE-MS and CAMMS223. Multiple sub-regions have reported on the use of ALZ in the treatment of multiple sclerosis. However, due to the duplication of numerous patients in these studies, repeatedly including them would lead to an increase in weight and potential bias within the study population. Therefore, we conducted extensive work to include only the studies in this series with later publication date and more complete data for statistical analysis (Figure 3).

### 3.2 Results and analysis

The study involved a total of 1729 patients, with 37 individuals experiencing ITP. In addition, 25 patients exhibited abnormal fluctuations in platelet levels (see details in the following text). Furthermore, 216 patients experienced autoimmune events, including three studies where it was not explicitly mentioned. The NOS scores for all included studies were greater than 5 points, indicating superior study quality. Fundamental information for the included studies is provided in the Supplementary Table 1.

The raw data for secondary ITP exhibited a normal distribution, so it was used for meta-analysis. The overall rate obtained was 0.0243 ([0.0176; 0.0321]) (*I*^2^ = 0%, *p* = 0.88). The Cochrane Q test and *I*^2^ test indicated no heterogeneity among the studies. The funnel plot and Egger’s test (*p* = 0.8424>0.05) revealed no significant publication bias. The sensitivity analysis conducted using the sequential omission method did not identify any studies that significantly influenced the results. Therefore, a fixed-effect model was used to describe the combined results, and the subgroup analysis is described in the following sections.

**Figure 4:**
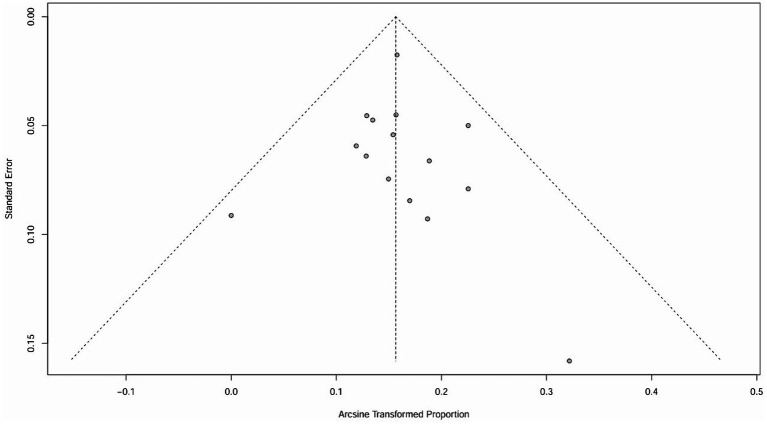
Funnel plot of ITP.

**Figure 5:**
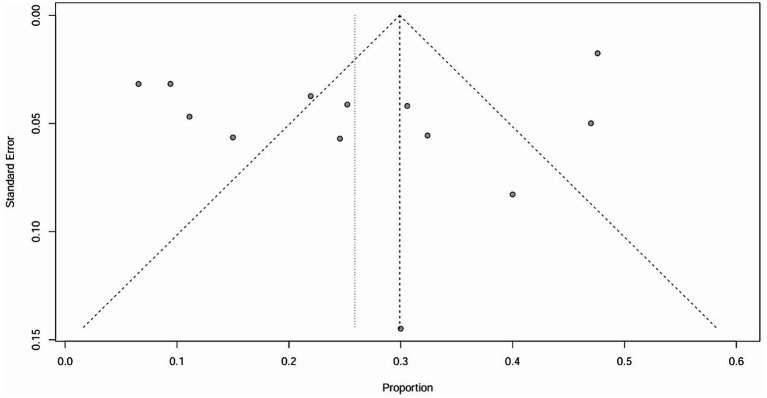
Funnel plot of ITP.

The raw data for secondary autoimmune events (AEs) demonstrated a normal distribution, thus it was also used for meta-analysis. The overall rate obtained was 0.2589 [0.1809; 0.3370] (*I*^2^ = 95%, *p* < 0.01). Statistically significant outliers (*p* < 0.05) were observed within the AEs. Visual inspection of the funnel plot and Egger’s regression intercept test indicated no statistically significant small study effects (*p* = 0.2035>0.05). Sensitivity analysis revealed a significant reduction in heterogeneity after the removal of one study, with an overall change of 0.0197 (6%). However, cumulative analysis demonstrated that since the publication of the first study in 2012, the incidence of AEs still exhibited statistical significance (Figures 4, 5).

It is important to note that the funnel plot, Egger’s test, and Begg’s test are methods used to assess publication bias. The funnel plot can be identified intuitively, whereas the other two methods involve quantitative detection. Egger’s test exhibits higher detection efficiency compared to Begg’s test. A *p*-value greater than 0.05 suggests no significant publication bias, while a value below or equal to 0.05 indicates significant publication bias (31). The results of Egger’s test in this study are all greater than 0.05, indicating that there is no significant publication bias.

### 3.3 Subgroup analysis

Two subgroup analyses were conducted in this study: one based on regional differences and the other based on different study types. The geographical coverage of the studies is broad, with nine studies conducted in Europe, four in North America, one in Oceania, and the remaining studies spanning multiple regions. Due to the fact that the number of studies for the latter two categories was ≤2, forest plots were not constructed, hence analysis was only conducted on Europe and North America. Based on regional subgroup analysis, the estimated variances for ITP in the European population are 2.07% [0.0110; 0.0334] (*I*^2^ = 0%, *p* = 0.80), while in North America, it is 3.52% [0.0185; 0.0667] (*I*^2^ = 0%, *p* = 0.62). Their interaction yields *Q* = 1.82, d.f. = 3, and *p* = 0.6109. Corresponding values for AEs are 21.58% [0.1367; 0.2948] (*I*^2^ = 85%, *p* < 0.01) and 26.22% [0.1274; 0.4639] (*I*^2^ = 90%, *p* < 0.01), with *Q* = 9.37, d.f. = 2, *p* = 0.0092. In subgroup analysis based on study type, the estimated incidence rates for ITP in prospective studies are 3.91% [0.0238; 0.0641] (*I*^2^ = 0%, *p* = 0.88), while in retrospective studies, it is 2.16% [0.0144; 0.0302] (*I*^2^ = 0%, *p* = 0.74), with an interaction value of *Q* = 1.72, d.f. = 1, *p* = 0.1902. For AEs, these values are 17.71% [0.1031; 0.3042] (*I*^2^ = 88%, *p* < 0.01) and 27.43 [0.2339; 0.3148] (*I*^2^ = 35%, *p* = 0.19), with an interaction result of *Q* = 21.02, d.f. = 1, *p* < 0.0001.

## 4 Discussion

The study Results showed that RRMS patients who received ALZ experienced a secondary ITP incidence rate of 2.43% and an AEs incidence rate of 25.89%. Clearly, the incidence rate of secondary diseases has increased compared to the normal population (32). Orla Tuohy et al. conducted a study in the UK from 1999 to 2012 with a sample size of 97 individuals, which revealed a secondary ITP incidence rate of 3.5% (3 cases) after ALZ treatment for RRMS (33). This finding differs from our study. Similarly, they obtained a 48% incidence rate of secondary immune-mediated disease, which may be attributed to their longer follow-up time. They followed the patients for a median of 7 years, with the longest follow-up period reaching 144 months. However, it should be noted that although a higher incidence of AID is observed, it cannot be attributed solely to ALZ. Longer follow-up times introduce higher uncontrollable confounding factors. Moreover, the relationship between MS and AID is still unclear and there is a lack of corresponding control studies. Therefore, based on these factors, it is not sufficient to determine which result is more convincing based solely on the length of follow-up.

In the included studies, Bachmann H et al. suggested that the thrombocytopenia related was not associated with ALZ intervention (24). However, they noted in their study that twenty out of thirty patients experienced mild or moderate reductions in platelet levels, all of which occurred during the first treatment cycle, and no bleeding symptoms were observed. Importantly, absolute platelet counts remained within the normal range in all patients. It is worth noting that this phenomenon occurred more than once. In all the included literature, three studies reported transient and temporary decreases in platelet levels, which returned to normal without intervention, and this accounted for a significant proportion (6, 24, 30). This is an intriguing observation that reminds us that ALZ may have a more pronounced interference with platelets and prompts us to consider the pathological mechanisms of ALZ treatment. Whether the abnormal fluctuations in platelet levels are merely transient changes after medication or an early indicator of secondary ITP, the underlying mechanisms of platelet fluctuations under ALZ intervention, and whether changes in platelet levels can be used as prognostic markers to monitor treatment efficacy after intervention warrants further exploration.

The exact etiology of MS remains unclear, posing a significant challenge for neurologists and rheumatologists in its treatment. Current research suggests that MS is an autoimmune disease in which the immune system mistakenly attacks its own tissues. B lymphocytes and T lymphocytes in the immune system are abnormally activated, leading to the production of Th cell cytokines and autoantibodies. These components erroneously target the myelin sheath, disrupting or interrupting the transmission of electric neural signals and triggering the associated symptoms. ALZ is a selective CD52 monoclonal antibody CD52 antigen is expressed on the surface of B cells, T cells, NK cells, most monocytes, and some granulocytes (34). It selectively binds to antigens on the surface of T lymphocytes and B lymphocytes, leading to lymphocyte and antibody-mediated lysis. After lymphocyte binding and depletion by ALZ, lymphocyte regeneration occurs, typically resulting in a recovery of B lymphocyte counts to baseline levels after 6 months of treatment, whereas T lymphocytes may take closer to 12 months to recover.

Treg cells, also known as regulatory T cells, are a subset of T lymphocytes that play a crucial role in immune regulation in the body, such as maintaining self-immune tolerance and preventing immune-mediated damage. MS is an autoimmune disorder characterized by immune dysregulation, and research has shown that the levels of Treg cells decrease significantly in MS patients within a week after the administration of ALZ. Interestingly, Haas J found that similar changes in Treg levels have also been observed in patients with ITP, occurring precisely within a week after the administration of ALZ (35). The immunological mechanisms of ITP have been extensively studied over the last decade, and a crucial factor identified is the deficiency of peripheral Tregs (36). Tregs are a subset of T cells labeled with CD4 + CD25hiFoxp3+, that make up approximately 5–10% of peripheral CD4 T cells and play a crucial role in self-tolerance (37). To explore the reasons for the deficiency of Tregs in ITP, Aslam R et al. studied an active ITP mouse model and discovered a different potential mechanism for the peripheral Treg defect in ITP, which involves the retention of Tregs in the thymus, leading to the loss of peripheral tolerance and allowing for the occurrence of immune responses against platelets (36). During the active phase of ITP, the deficiency of peripheral Tregs may be a result of the isolation of functional Tregs in the thymus. After normalization of platelet counts with IVIg treatment, a significant reduction in thymic Tregs and rescue of the peripheral Treg pools in the was observed. Subsequently, an increase in peripheral Treg cells was associated with an improvement in platelet levels (38). This increase is likely attributed to the replenishment of Treg cells from the central thymus (36). Similarly, following the administration of ALZ, a notable decrease in Treg levels was observed. ALZ exerts its inhibitory effect on autoimmune attacks by depleting self-reactive T cells and B cells. After receiving anti-CD52 treatment, the number of peripheral T cells, including Treg cells, decreases (36). This indicates a clear interference of ALZ with Treg cells and is consistent with the changes observed in Treg levels in ITP patients (39). Based on the shared changes in Treg cell levels, it can be inferred that Treg cells may be one of the mechanisms by which ALZ modulates the cellular and subsequent platelet level changes in MS. Therefore, in clinical practice, assessing the thymic function of MS patients in advance may help evaluate and predict the secondary ITP prognosis after ALZ intervention. However, this is a theoretical speculation and the study conducted by Haas et al. had a relatively small sample size of 15 participants (35). Therefore, further studies with larger sample sizes are still needed for analysis, exploration and validation. Furthermore, Tobias Ruck et al. (40) found in a study conducted in 2022 that patients with MS who developed secondary autoimmune disorder (SAID) after ALZ treatment also exhibited an excessive expansion of T cell clones at baseline. This may have predictive implications for the development of SAID in individuals undergoing ALZ therapy, with the remaining T cell repertoire being closely associated with the occurrence of autoimmunity. And Heinz Wiendl et al. (41) also demonstrated through lymphocyte phenotyping and pharmacodynamic assessment that the kinetic of peripheral lymphocyte subpopulations proliferation in MS patients receiving ALZ intervention did not predict the occurrence or activity of autoimmune diseases. These findings suggest that that quality of the repertoire might be more relevant than the dynamic of repopulation.

Treg cells have significant anti-inflammatory effects and can secrete anti-inflammatory cytokines such as IL-4, IL-10, TGF-β to inhibit self-inflammatory responses, preventing pathological immune responses that can cause tissue damage. However, they are also responsible for the difficulty in clearing pathogens in cases of long-term infection, as Treg cells prolong the course of chronic infection. Defective or deficient Treg cell function directly leads to the development of inflammatory diseases and plays an essential role in numerous chronic inflammatory diseases (42). Platelets have long been recognized as immune cells that promote leukocyte and endothelial activation, stimulate neutrophil extracellular trap formation, detect and clear pathogens, and promote inflammatory responses. The inhibitory effect of ALZ intervention on inflammation is antagonistic to the role of platelets in the body, but the mechanism by which ALZ interferes with platelet levels needs further investigation.

The monoclonal antibody ALZ induces persistent and significant depletion of circulating T and B lymphocytes through complement and antibody-dependent cell cytotoxicity. No significant inhibitory effect on the bone marrow has been observed (7). Current research also suggests that ALZ induced changes in platelet levels may occur during platelet destruction. However, it cannot be ruled out that ALZ may interfere with the process of platelet generation. Future experiments can aim to identify whether ALZ interferes with platelet generation or destruction by increasing the detection of absolute immature platelet fraction (IPF) as a diagnostic indicator. This will help in better understanding the underlying mechanisms of secondary ITP (43).

Vigilant monitoring of platelets in MS patients following ALZ administration is imperative. Regular hematology surveillance, for example, facilitates better adherence to treatment. The U.S. Alemtuzumab Risk Evaluation and Mitigation Strategy (REMS), as well as Risk Management Plans (RMP) in the European Union and other countries worldwide, recommend monitoring from initiation of treatment until 48 months after the last alemtuzumab infusion. The majority of secondary ITP cases are identified through risk monitoring and a minority are identified through symptoms and signs. Diagnosis can be established on the basis of abnormal platelet count levels, followed by necessary action and proactive treatment after confirmation (44). Treatment of secondary ITP is generally aligned with standard protocols. First-line therapy consists of steroid use with or without intravenous immunoglobulin and/or platelet transfusions, while second-line therapy primarily involves rituximab. Studies have shown that secondary ITP following ALZ therapy in adults often exhibits a shorter duration and greater responsiveness to conventional therapy compared to primary ITP, resulting in effective remission for most patients after treatment. Therefore, regular and timely vigilant monitoring during the application of ALZ is crucial for early detection, appropriate management and disease reduction (45, 46).

We performed a subgroup analysis of the 15 papers included in the study that met the predefined grouping criteria, mainly including regional and study type analysis. The results show that the incidence of secondary diseases is not the same in different regions, suggesting that there are significant regional differences in the occurrence of secondary diseases, which may be related to human lifestyle, medical conditions, dietary concepts, and physique. It should be noted that there was heterogeneity in the analysis of adverse event incidence in both regions, and factors that affected the results of the regional subgroup analysis could also be potential sources of heterogeneity.

Subgroup analyses for different study types also revealed significant differences in results, which may be due to the fact that prospective studies design their protocols and data collection instruments prior to including patients, making them less susceptible to interference factors than retrospective studies. As a result, the results of prospective studies may be closer to the true incidence rate than retrospective analyses. We look forward to larger sample sizes and better designed prospective clinical studies in the future.

### 4.1 Limitations

This systematic review has several limitations. Firstly, it is crucial to consider whether the increased incidence of secondary ITP and AEs is due to the effect of ALZ or if there is a higher rate of ITP or immune-related disease occurrence in patients with MS itself. We need to investigate whether there is a difference in the proportion of secondary diseases between MS patients treated with ALZ and those not treated. In addition, we need to determine if there is a higher rate of adverse events associated with the use of ALZ compared to other medications in MS patients. Comparative studies involving multiple interventions are still limited and obtaining accurate data on these issues remains challenging. This suggests the need for further research to compare and analyze these aspects for more accurate information. Furthermore, there is a lack of a gold standard diagnostic test for ITP, and the assessment of the severity of the disease using biomarkers is still in its early stages (38). The included studies do not provide specific information on platelet level fluctuations, making it difficult to determine the severity of platelet reduction. This indicates the importance for researchers in future studies to provide more detailed grouping descriptions in order to obtain more precise and accurate data. It should be noted that the term ITP, which stands for Idiopathic Thrombocytopenic Purpura, was renamed Immune Thrombocytopenia by the International Working Group on ITP in 2007, and that all of the trials designed with ALZ included in this study were conducted after 2016, thus excluding any conceptual confusion caused by the term ITP. Each study was not entirely consistent in terms of pre-medication protocols. In order to mitigate adverse reactions such as infusion reactions caused by rapid depletion of the immune system, a combination of multiple drugs is often used, and various preventive protocols have been established. However, the optimal dosage and timing of these drugs are still unclear. Pre-medication options include corticosteroids, antihistamines, or antiviral drugs, and the types and dosages of drugs used are not entirely uniform, which may be a source of heterogeneity. The included studies had relatively long follow-up periods, and individual patients, particularly those with the disease, could be affected by a variety of factors that could affect the accuracy of the data. This is an important consideration. Considering these factors, the strength of the evidence regarding the impact of ALZ on the incidence of secondary adverse events in MS patients may be diminished.

### 4.2 Strengths

Given the current high prevalence of MS and the challenges associated with its treatment, various real-world studies have been conducted in recent years by different countries. However, a systematic review and meta-analysis regarding the occurrence of ITP events after the administration of ALZ for the treatment of MS has not been previously identified. This study represents the first analysis of the incidence of secondary ITP in MS patients following the use of ALZ, while also providing a statistical assessment of adverse events occurrence. Furthermore, speculation has been made regarding the potential underlying mechanisms. Drawing on high-quality real-world clinical research literature published over the past decade, this study contributes robust additional data evidence.

## 5 Conclusion

Secondary ITP is a recognized adverse event following the use of ALZ intervention for MS. The results of data analysis demonstrate that ALZ affects platelet levels in MS patients, leading to a certain incidence of ITP. This phenomenon may be associated with a common mechanism in the development of ITP, particularly involving Treg cells. The role of cytotoxic T cell s and inflammatory responses may also contribute significantly to the induction of ITP. These findings highlight the importance of conducting further in-depth analyses and extending the duration of follow-up observations to gain more precise and detailed evidence regarding the efficacy of ALZ therapy for managing changes in platelet levels in MS patients. Exploring the specific pathological mechanisms involved is crucial in advancing our understanding in this area. Additionally, these results underscore the importance of conducting a comprehensive evaluation of the potential benefits and risks of ALZ treatment in MS patients in clinical practice. Factors such as age, economic status, physical health, and susceptibility to secondary diseases should be carefully considered when developing personalized treatment plans to optimize clinical management and enhance patient outcomes.

## Data availability statement

The original contributions presented in the study are publicly available. This data can be found here: https://data.mendeley.com/datasets/dpj8rvx68r/1.

## Author contributions

YS: Conceptualization, Writing – original draft. ZL: Writing – review & editing. JY: Conceptualization, Methodology, Writing – review & editing. QJ: Data curation, Supervision, Writing – review & editing. JS: Data curation, Supervision, Writing – review & editing. LW: Formal analysis, Writing – review & editing. FL: Formal analysis, Writing – review & editing. SS: Investigation, Writing – review & editing. KW: Project administration, Writing – review & editing. XZ: Funding acquisition, Resources, Writing – review & editing.

## Footnotes

^1^ http://www.ohri.ca/programs/clinical_epidemiology/oxford.asp

## Glossary

MS: multiple sclerosis
ALZ: alemtuzumab
ITP: immune thrombocytopenia
IMT: Immunomodulatory Therapies
FDA: Food and Drug Administration
EMA: European Medicines Agency
SAID: secondary autoimmune disorder
IFN-b: beta-interferon
NOS: Newcastle-Ottawa Scale
AEs: Autoimmune events
RRMS: remitting–relapsing multiple sclerosis,
CI: confidence interval
IPF: immature platelet fraction
ATEs: autoimmune thyroid events
CLL: chronic lymphocytic leukemia
REMS: Risk Evaluation and Mitigation Strategy
RMP: Risk Management Plans
