# Supplementary material for: Risk of secondary immune thrombocytopenia following alemtuzumab treatment for multiple sclerosis: a systematic review and meta-analysis
Source: Front Neurol. 2024 Apr 10;15:1375615. doi: 10.3389/fneur.2024.1375615 (PMC11039963; doi:10.3389/fneur.2024.1375615)
Supplement: Supplementary file 1 [file Table_1.docx]

|  | selection | | | | | | | | | | | | | Comparability | | outcome | | | | | | | | | |  |
| --- | --- | --- | --- | --- | --- | --- | --- | --- | --- | --- | --- | --- | --- | --- | --- | --- | --- | --- | --- | --- | --- | --- | --- | --- | --- | --- |
| name | 1） | | | | 2） | | | 3） | | | | 4） | | 5） | | 6） | | | | 7) | | 8) | | | | score |
|  | 1a) | 1b) | 1c) | 1d) | 2a) | 2b) | 2c) | 3a) | 3b) | 3c) | 3d) | 4a) | 4b) | 5a) | 5b) | 6a) | 6b) | 6c) | 6d) | 7a) | 7b) | 8a) | 8b) | 8c) | 8d) |  |
| Bose  2021 |  | ● |  |  | ● |  |  | ● |  |  |  | ● |  | ● |  |  | ● |  |  | ● |  | ● |  |  |  | 8 |
| Fox  2012 |  | ● |  |  |  |  |  |  | ● |  |  | ● |  | ● |  |  | ● |  |  | ● |  | ● |  |  |  | 7 |
| Rauma 2022 | ● |  |  |  |  |  |  | ● |  |  |  | ● |  | ● |  |  | ● |  |  | ● |  | ● |  |  |  | 7 |
| Vukusic 2019 | ● |  |  |  |  |  |  |  | ● |  |  | ● |  | ● |  |  | ● |  |  |  | ● |  | ● |  |  | 6 |
| Eichau 2023 |  | ● |  |  |  |  |  | ● |  |  |  | ● |  | ● |  |  | ● |  |  | ● |  | ● |  |  |  | 7 |
| Keller1 2019 |  | ● |  |  |  |  |  |  | ● |  |  | ● |  | ● |  |  | ● | ● |  | ● |  | ● |  |  |  | 7 |
| Leckey 2022 |  | ● |  |  |  |  |  |  | ● |  |  | ● |  | ● |  |  | ● | ● |  |  | ● |  | ● |  |  | 6 |
| Häußler 2021 |  | ● |  |  | ● |  |  | ● |  |  |  | ● |  | ● |  |  | ● | ● |  | ● |  | ● |  |  |  | 8 |
| Willis 2016 | ● |  |  |  |  |  |  |  | ● |  |  | ● |  | ● |  |  | ● | ● |  | ● |  |  | ● |  |  | 7 |
| Boffa 2020 |  | ● |  |  | ● |  |  |  | ● |  |  | ● |  | ● |  |  | ● |  |  | ● |  | ● |  |  |  | 8 |
| Bachmann 2021 |  | ● |  |  |  |  |  | ● |  |  |  | ● |  | ● |  |  | ● |  |  | ● |  |  | ● |  |  | 7 |
| di Ioia 2020 |  | ● |  |  |  |  |  | ● |  |  |  | ● |  | ● |  |  | ● |  |  | ● |  |  |  |  | ● | 6 |
| Brecl Jakob 2021 | ● |  |  |  |  |  |  | ● |  |  |  | ● |  | ● |  |  | ● |  |  | ● |  | ● |  |  |  | 7 |
| Reddel, 2019 |  | ● |  |  |  |  |  |  | ● |  |  | ● |  | ● |  |  | ● |  |  | ● |  | ● |  |  |  | 7 |
| Bass  2021 |  | ● |  |  |  |  |  | ● |  |  |  | ● |  | ● |  |  | ● |  |  | ● |  |  | ● |  |  | 7 |

1) Representativeness of the exposed cohort; 2) Selection of the non exposed cohort; 3) Ascertainment of exposure; 4) Demonstration that outcome of interest was not present at start study; 5) Comparability of cohorts on the basis of the design or analysis; 6) Assessment of outcome; 7) Was follow-up long enough for outcomes to occur; 8) Adequacy of follow up of cohorts. 1a) Truly representatively of the average in the community; 1b) somewhat representative of the average in the community; 1c) selected group of users, for example, nurses, volunteers; 1d) no description of the derivation of the cohort; 2a) drawn from the same community as the exposed cohort; 2b) drawn from a different source; 2c) no description of the derivation of the non exposed cohort; 3a) secure record (e.g. surgical records); 3b) structures interview; 3c) written self report; 3d) no description; 4a) yes; 4b) no; 5a) study controls for the most important factor; 5b) study control for any additional factor (This criteria could be modified to indicate specific control for a second important factor.); 6a) independent blind assessment; 6b) record linkage; 6c) self report; 6d) no description; 7a) the follow-up time was ≥24 months; 7b) the follow-up time was ＜24 months; 8a) complete follow up all subjects accounts for; 8b) few subject lost to follow up unlikely to introduce bias (the loss to follow-up situation has been described.); 8c) follow up rate were described and no description of those lost; 8d) no statement.
