# Supplementary material for: Risk of secondary immune thrombocytopenia following alemtuzumab treatment for multiple sclerosis: a systematic review and meta-analysis
Source: Front Neurol. 2024 Apr 10;15:1375615. doi: 10.3389/fneur.2024.1375615 (PMC11039963; doi:10.3389/fneur.2024.1375615)
Supplement: Supplementary file 2 [file Data_Sheet_1.docx]

**Detailed search strategy in each database**

**1.PubMed（n=55）：**

((Idiopathic Thrombocytopenic Purpura) OR (Idiopathic Thrombocytopenic Purpuras) OR (Purpura, Idiopathic Thrombocytopenic) OR (Purpuras, Idiopathic Thrombocytopenic) OR (Thrombocytopenic Purpura, Idiopathic) OR (Thrombocytopenic Purpuras, Idiopathic) OR (Immune Thrombocytopenic Purpura) OR (Immune Thrombocytopenic Purpuras) OR (Purpura, Immune Thrombocytopenic) OR (Purpuras, Immune Thrombocytopenic) OR (Thrombocytopenic Purpura, Immune) OR (Thrombocytopenic Purpuras, Immune) OR (Immune Thrombocytopenia) OR (Immune Thrombocytopenias) OR (Thrombocytopenia, Immune) OR (Thrombocytopenias, Immune) OR (Thrombocytopenic Purpura, Autoimmune) OR (Werlhof Disease) OR (Disease, Werlhof) OR (Werlhof's Disease) OR (Disease, Werlhof's) OR (Werlhofs Disease) OR (Autoimmune Thrombocytopenia) OR (Autoimmune Thrombocytopenias) OR (Thrombocytopenia, Autoimmune) OR (Thrombocytopenias, Autoimmune) OR (Autoimmune Thrombocytopenic Purpura) OR (Autoimmune Thrombocytopenic Purpuras) OR (Purpura, Autoimmune Thrombocytopenic) OR (Purpuras, Autoimmune Thrombocytopenic) OR (Purpura, Thrombocytopenic, Autoimmune)) AND ((Sclerosis, Multiple) OR (Sclerosis, Disseminated) OR (Disseminated Sclerosis) OR (MS (Multiple Sclerosis)) OR (Multiple Sclerosis, Acute Fulminating)) AND ((Alemtuzumab) OR (Campath 1H) OR (Monoclonal Antibody Campath-1H) OR (Antibody Campath-1H, Monoclonal) OR (Campath-1H, Monoclonal Antibody) OR (Monoclonal Antibody Campath 1H) OR (Campath-1H) OR (Campath1H) OR (Campath 1M) OR (Campath-1M) OR (MabCambath) OR (Lemtrada) OR (Campath 1G) OR (Campath-1-G) OR (Campath 1 G) OR (Campath1G) OR (Campath-1G) OR (Campath))

**2.OVID（n=343）：**

Books@Ovid <May 01, 2023>

EBM Reviews - Cochrane Database of Systematic Reviews <2005 to May 2, 2023>

EBM Reviews - ACP Journal Club <1991 to April 2023>

EBM Reviews - Database of Abstracts of Reviews of Effects <1st Quarter 2016>

EBM Reviews - Cochrane Clinical Answers <April 2023>

EBM Reviews - Cochrane Central Register of Controlled Trials <April 2023>

EBM Reviews - Cochrane Methodology Register <3rd Quarter 2012>

EBM Reviews - Health Technology Assessment <4th Quarter 2016>

EBM Reviews - NHS Economic Evaluation Database <1st Quarter 2016>

Embase <1974 to 2023 May 04>

JBI EBP Database <Current to April 26, 2023>

Journals@Ovid Full Text and abstracts

Journals@Ovid held by WOHS

Ovid Healthstar <1966 to March 2023>

Ovid MEDLINE(R) ALL <1946 to May 04, 2023>

APA PsycInfo <1806 to April Week 4 2023>

**3.EMBASE（n=350）：**

(alemtuzumab)/br AND (('multiple sclerosis')/br) AND (('autoimmune thrombocytopenia')/br)

**4.WOS（n=40）：**

((ALL=(Alemtuzumab)) AND ALL=(Multiple Sclerosis)) AND ALL=(immune thrombocytopenia)
